# The Emergence of Visual Awareness: Temporal Dynamics in Relation to Task and Mask Type

**DOI:** 10.3389/fpsyg.2017.00315

**Published:** 2017-03-03

**Authors:** Markus Kiefer, Thomas Kammer

**Affiliations:** ^1^Section for Cognitive Electrophysiology, Department of Psychiatry, Ulm UniversityUlm, Germany; ^2^Section for Neurostimulation, Department of Psychiatry, Ulm UniversityUlm, Germany

**Keywords:** visual masking, awareness, consciousness, psychophysics, vision, graded, dichotomous

## Abstract

One aspect of consciousness phenomena, the temporal emergence of visual awareness, has been subject of a controversial debate. How can visual awareness, that is the experiential quality of visual stimuli, be characterized best? Is there a sharp discontinuous or dichotomous transition between unaware and fully aware states, or does awareness emerge gradually encompassing intermediate states? Previous studies yielded conflicting results and supported both dichotomous and gradual views. It is well conceivable that these conflicting results are more than noise, but reflect the dynamic nature of the temporal emergence of visual awareness. Using a psychophysical approach, the present research tested whether the emergence of visual awareness is context-dependent with a temporal two-alternative forced choice task. During backward masking of word targets, it was assessed whether the relative temporal sequence of stimulus thresholds is modulated by the task (stimulus presence, letter case, lexical decision, and semantic category) and by mask type. Four masks with different similarity to the target features were created. Psychophysical functions were then fitted to the accuracy data in the different task conditions as a function of the stimulus mask SOA in order to determine the inflection point (conscious threshold of each feature) and slope of the psychophysical function (transition from unaware to aware within each feature). Depending on feature-mask similarity, thresholds in the different tasks were highly dispersed suggesting a graded transition from unawareness to awareness or had less differentiated thresholds indicating that clusters of features probed by the tasks quite simultaneously contribute to the percept. The latter observation, although not compatible with the notion of a sharp all-or-none transition between unaware and aware states, suggests a less gradual or more discontinuous emergence of awareness. Analyses of slopes of the fitted psychophysical functions also indicated that the emergence of awareness of single features is variable and might be influenced by the continuity of the feature dimensions. The present work thus suggests that the emergence of awareness is neither purely gradual nor dichotomous, but highly dynamic depending on the task and mask type.

## Introduction

Elucidating the cognitive and neural mechanisms underlying phenomenal consciousness (Block, 1995), that is, the experiential qualities of sensations, remains one of the greatest and most exciting scientific endeavors in the 21st century. One particular challenge in the scientific explanation of phenomenal consciousness is the privacy of subjective experiences (Nagel, 1974), which renders an objective assessment of the phenomenon in question difficult. However, an adequate characterization of consciousness phenomena is an important prerequisite for determining the underlying neuro-cognitive mechanisms (Dehaene and Naccache, 2001; Kiefer et al., 2011).

One aspect of consciousness phenomena, the temporal emergence of visual awareness, has been subject of a controversial debate (for a review see, Windey and Cleeremans, 2015). A first class of proposals assumes that visual awareness is a phenomenon gradually developing over time ranging from unawareness over a coarse glimpse to full awareness of the visual stimulus (Dennett and Kinsbourne, 1992; Overgaard et al., 2006; Seth et al., 2008). A second opposing class of proposals argues that consciousness is an all-or-none phenomenon (Sergent and Dehaene, 2004; Quiroga et al., 2008; Sekar et al., 2013; Asplund et al., 2014). These proposals state that the transition between unawareness and full awareness of a stimulus proceeds in a binary fashion. A third class of intermediate proposals suggests independently accessible levels of representation such as physical energy, simple visual features, letters, word forms and meaning (Kouider et al., 2011; Windey et al., 2013; Windey and Cleeremans, 2015). States of full awareness include access to all levels of representations, whereas in complete unawareness there is no access to any level. Most critically, this class of proposals also assumes states of partial awareness, in which observers only experience the informational content of some restricted levels. According to different variants of this partial awareness hypothesis, access to the different levels of representation either exclusively occurs in an all-or-none fashion (Kouider et al., 2011) or might depend on the feature type: Conscious access to low-level visual features (such as energy, geometrical elements, lines and color) is assumed to occur in a gradual fashion, whereas access to higher-level features (e.g., word form or meaning) is supposed to be all-or-none (Windey et al., 2013; Windey and Cleeremans, 2015). Higher-level features of visual stimuli referring to lexical (word form) or semantic representations (meaning) transcendent the information provided by the visual sensory channel and can be characterized as multimodal or even amodal in some aspects (Kiefer and Pulvermüller, 2012). In keeping the terminology with previous research and for simplicity reasons, we use the term “visual awareness” throughout this paper also in the context of these higher-level features, in order to express that their representations are accessed from visual stimuli.

Most previous studies addressing the temporal emergence of visual awareness used subjective awareness ratings of the clarity of the percept as primary variable of interest (e.g., Sergent and Dehaene, 2004; Overgaard et al., 2006; Windey et al., 2013). Such subjective measures based on introspection were preferred over objective measurements of discrimination performance based on signal detection theory, because the latter measures can also capture the influence of unconscious processing and cannot be taken as an exclusive index of visual awareness (Sandberg et al., 2011). Furthermore, as objective awareness measures based on signal detection theory typically average detection performance over a series of trials, they do not consider the entire performance distribution. However, the validity of subjective ratings of introspective experience in single trials has also been controversially discussed: Subjective ratings might be affected by response biases and cannot be necessarily taken as direct reflections of subjective visual experience (Snodgrass and Shevrin, 2006; Asplund et al., 2014; Schmidt, 2015).

In addition to these measurement problems, previous studies yielded heterogeneous results (for a discussion see, Bachmann, 2013) supporting all-or-none (Sergent and Dehaene, 2004; Quiroga et al., 2008; Sekar et al., 2013; Asplund et al., 2014), graded (Overgaard et al., 2006; Sandberg et al., 2010), or partial-awareness proposals (Windey et al., 2013). Given that different experimental blinding methods (visual masking vs. attentional blink), different stimuli or stimulus features (low level vs. high level) or masks (random pattern vs. random letters) were used in the previous studies, it is well conceivable that these conflicting results are more than noise, but reflect the dynamic and context-dependent nature of the temporal emergence of visual awareness (see also, Overgaard and Mogensen, 2016). We propose that, depending on the specific stimulation context and blinding method, the percept could emerge either in an all-or-none or in a more graded fashion. Despite some differences (for a discussion see, Breitmeyer et al., 2015), many current models converge on the assumption that visual awareness requires recurrent processing of the stimulus within multiple brain systems, thereby consolidating its representation (Enns and Di Lollo, 2000; Dehaene and Naccache, 2001; Lamme, 2003; Kiefer et al., 2011). Consolidation through recurrent processing can be characterized as reaching an attractor state within neural networks (Herzog et al., 2016). Depending on the specific context, the attractor state might be reached from previous intermediate states of unconscious processing through relatively sharp or smooth transitions resulting in a more dichotomous or gradual emergence of awareness. Furthermore, again depending on the context, the attractor state might encompass different neural systems coding specific features of the stimulus. This would lead to awareness of just a few or all stimulus features as suggested by the partial awareness hypothesis (Kouider et al., 2011).

In line with such a dynamic and context-dependent view of the temporal emergence of visual awareness, findings with masking and attentional blink paradigms yielded quite heterogeneous results depending on the specific stimuli used. For instance, attentional blink paradigms with word, color or face stimuli produced a data pattern consistent with an all-or-none emergence of a visual percept (Sergent and Dehaene, 2004; Asplund et al., 2014), whereas attentional blink paradigms with letters were associated with a more gradual emergence of a visual percept (Nieuwenhuis and de Kleijn, 2011). Masking experiments with complex pattern masks, in contrast, frequently yielded results consistent with a gradual emergence of consciousness (Sergent and Dehaene, 2004; Sandberg et al., 2010).

In order to address the issue whether visual awareness emerges dynamically in a context-dependent fashion, in the present study, we determined identification and discrimination thresholds for stimulus features of varying complexity in different masking contexts using a temporal two-alternative forced choice task (temporal 2-AFC). This temporal 2-AFC task has the advantage to provide an objective psychophysical measurement of identification or discrimination performance as an index of visual awareness with a comparable set of word stimuli while minimizing biases from unconscious processing. In the task, participants were presented with two stimulus-mask sequences separated by a delay of 900 ms. The critical task-relevant stimulus feature (energy or stimulus presence/letter case/lexicality/semantics) randomly appeared either in the first or the second interval. After the second sequence, participants were prompted to indicate in which interval the designated stimulus feature was presented. Above-threshold performance in this temporal 2-AFC task most likely exclusively reflects awareness of the critical feature, because response biases from unconscious processing are minimized for several reasons: Semantic priming elicited by unconsciously perceived masked stimuli have been shown to decay rapidly after about 100 ms (Brown and Hagoort, 1993; Greenwald et al., 1996; Kiefer and Spitzer, 2000; Kiefer and Brendel, 2006), while response tendencies initiated by masked stimuli are even inhibited after this time interval (Eimer and Schlaghecken, 2003). As the response in this task is delayed after the second presentation interval, it is unlikely that unconscious semantic priming or visuo-motor processes were able to bias the response (see also, Milner and Dijkerman, 2001). Furthermore, the task requires the comparison of the percepts in intervals one and two. We are not aware of any evidence that such a complex comparison can be performed on the basis of unconscious visual processes (Ansorge et al., 2014).

Visibility of the word stimuli were manipulated by gradually varying the target stimulus mask onset asynchrony (stimulus mask SOA) according to a staircase algorithm. Psychophysical functions were then fitted to the accuracy data of stimulus detection (presence of an stimulus or energy) or discrimination (letter case/lexicality/semantics) performance in the different task conditions as a function of the stimulus mask SOA in order to determine the conscious detection or discrimination threshold (point of inflection of the logistic function, estimated accuracy of 75%). A higher threshold indicates that the stimulus has to be presented longer in isolation before mask onset so that a given task-relevant feature can be consciously identified.

In addition to thresholds, slopes of the fitted psychophysical functions in the different conditions can also be determined. A steep slope of the function is taken to index that visual awareness of a given feature emerges in a more all-or-none fashion, whereas a shallow slope is assumed to indicate a gradual transition from unconscious to conscious at the feature level (Koch and Preuschoff, 2007; Sandberg et al., 2011). Hence, when analyzing thresholds and slopes of the psychophysical function we can determine the relative timing of access to consciousness across stimulus features (thresholds) as well as the abruptness vs. smoothness of the transition from the unconscious to the conscious within stimulus features (slopes).

Our experimental approach using stimulus mask SOA as variable to infer the temporal emergence of awareness within the temporal 2AFC task is based on the following rationale: The higher the threshold in terms of stimulus mask SOA, the longer the stimulus requires processing within the visual system to achieve consolidation before the mask interferes with its processing. Based on these considerations, the stimulus mask SOA provides information about the approximate time point, at which masked stimulus features are sufficiently consolidated to be available for conscious access as indicated by above-threshold performance in the temporal 2AFC task (estimated SOA at 75% correct performance). Whether the observed time course of features thresholds not only reflects the time course of conscious access, but also the time course of phenomenal experience critically depends on the plausible assumption that visual awareness (subjective perceptual experience) and access consciousness (above-threshold discrimination performance of stimuli) exhibit an at least correlated time course. Furthermore, our psychophysical approach informs us about the temporal emergence of features only for briefly presented masked stimuli, and is mute with regard to the relative timing of stimulus features under unmasked conditions or for longer stimulus duration. However, these limitations apply to all experimental approaches to consciousness; whether the blinding technique of choice is a masking or attentional blink paradigm, or whether psychophysical measures such as thresholds and slopes or subjective awareness ratings are used as index of awareness.

Although psychophysical functions are necessarily fitted on performance data of a series of trials, the threshold or slope parameters of this function characterize the entire accuracy distribution across single trials with varying stimulus mask SOAs and are not simple average performance measures. Hence, the criticism of objective awareness measures based on averages across trials, as raised in the context of signal detection theory mentioned above, does not apply to our psychophysical approach.

The competing proposals of the temporal emergence of visual awareness make different predictions regarding the relative temporal ordering of thresholds for the different task-relevant stimulus features and slopes of the psychophysical functions. According to all-or-none proposals, thresholds of the different features should be very similar, and psychophysical functions should generally have a steep slope (Del Cul et al., 2009). Proposals assuming a gradual emergence of consciousness (Dennett and Kinsbourne, 1992; Overgaard et al., 2006; Seth et al., 2008) as well as the partial awareness hypothesis (Kouider et al., 2011; Windey et al., 2013; Windey and Cleeremans, 2015), in contrast, predict that threshold SOAs of the features should depend on their level of complexity with energy (stimulus presence decision) having the lowest threshold followed by letter form (capital letter decision), word form (lexical decision), and meaning (semantic decision).

Although all variants of the partial awareness hypothesis suggest a sequential contribution of stimulus features to the conscious percept as a function of their complexity level, there is a controversy how awareness of *one particular feature* emerges. According to one variant, the transition from unawareness to awareness for each feature occurs in an all-or-none fashion (Kouider et al., 2011). This implies that threshold SOAs for the features should show the temporal gradient described above, but the slope of the psychometric function should be invariantly steep. According to the other variant (Windey et al., 2013), transition from unawareness to awareness for the different features depends on their complexity level. Hence, this proposal predicts both a temporal gradient of threshold SOAs and a variation of slopes of the psychometric function: Awareness for low-level features such as color should emerge gradually resulting in a shallower slope of the psychometric function. Awareness for higher-level features such as semantics should emerge in an all-or-none fashion resulting in steep slopes.

### Predictions with Regard to Threshold SOAs in Relation to Mask Type and Task

Target-mask threshold SOAs indicate the time needed for visual consolidation so that a given stimulus feature is available for conscious access. In order to test the contextual dynamics of visual awareness, we created four different masks, which systematically varied with regard to their similarity with the word targets (random pattern mask, false font mask, random letter mask, and word mask). Masks are more efficient to suppress stimulus visibility, when mask and target share common features (Breitmeyer and Öğmen, 2006). Likewise, comparable similarity effects between targets and distractors have been observed within the context of the attentional blink paradigm (Maki et al., 2003; Dux and Coltheart, 2005). We therefore predicted that higher-level masks (random letters, word) should yield higher thresholds SOAs compared with low-level masks (random pattern and false fonts). Most critically, in line with the notion of a context-dependent dynamic emergence of visual awareness, as outlined above, we expected that the relative ordering of threshold SOAs in the different task conditions (stimulus presence, letter case, lexicality, and semantic category) is modulated by the type of mask: As the random pattern mask does not bear any similarity with any task-relevant word feature, all features should quite simultaneously contribute to the conscious percept. Threshold SOAs in the different tasks should be therefore similar or even identical indicating that the conscious percept emerges in a more discontinuous, or even in an all-or-none fashion. In contrast, for higher-level letter and word masks, which specifically interfere with the lexical or semantic levels of word representation, access to the corresponding features of the percept should be delayed compared with low-level features. We therefore expected to observe a greater differentiation of threshold SOAs in the different tasks with these higher-level masks. As a differentiation of thresholds indicates that features can be discriminated more sequentially at distinct stimulus mask SOAs, such a pattern of results would be consistent with a more gradual emergence of visual awareness.

### Predictions with Regard to the Slopes of the Psychophysical Functions in Relation to Mask Type and Task

The slopes of the psychophysical function index the transition from unawareness to awareness within each task probing a specific feature type (Koch and Preuschoff, 2007; Windey et al., 2013). All-or-none proposals (e.g., Del Cul et al., 2009) predict generally steep slopes (and comparable SOA thresholds for all tasks). The analysis of slopes is suited to distinguish between different variants of the partial awareness hypotheses, which all predict a temporal gradient of threshold SOAs as outlined above. If conscious access to individual features were generally dichotomous as assumed in the framework by Kouider et al. (2011), slopes of the psychophysical function in the different tasks should be invariantly steep. In contrast, the partial awareness framework proposed by Windey et al. (2013) and Windey and Cleeremans (2015) predicts shallower slopes, i.e., more gradual emergence of awareness, for low-level visual features (energy, letter form) compared with higher-level word form or semantic features, which should exhibit a more discontinuous emergence of awareness. However, a further scenario is also conceivable: The complexity of processing and thus the time needed for consolidation may also influence the transition from unawareness to awareness for individual features, resulting in shallower slopes for features with longer threshold SOAs. As described above, random letter and word masks may specifically interfere with and thus delay the processing of higher-level lexical and semantic features compared with the more neutral pattern and false font masks. As a consequence, for higher-level features the transition from unawareness to awareness would be more gradual under random letter and word masks resulting in shallower slopes in the lexical and semantic tasks.

## Materials and Methods

### Subjects

Eighty-four subjects (mean age 22.3, 47 female) were recruited for the study. They were native German-speaking volunteers without any history of neurological or psychiatric disorders. They participated after giving written informed consent and they were compensated for participation either by money or by course credits. The study has been approved by the institutional review board of Ulm University. Sample size was determined according to earlier psychophysical studies. The effects of the four different masks were investigated in a between-subject design (see below). Each subject was randomly assigned to one of the four groups. Data collection was stopped when the data of at least 16 subjects within each group fulfilled the inclusion criteria for analysis (see below).

Visual acuity was tested using FrACT (Version 3.7.1b, central Landoldt-C, 4AFC, 30 trials, observer distance 2.5 m, Bach, 1996). All subjects had a binocular visuals of 0.85 at minimum, and no subject was excluded due to vision impairment.

### Stimuli and Apparatus

Five word lists comprising 50 pairs of stimuli were generated from the CELEX lexical database (Baayen et al., 1995). Only words with six characters were used. For the different tasks (see below) different word lists were used (see Supplementary Table S1), which were matched for word frequency and word length.

**FIGURE 1:**
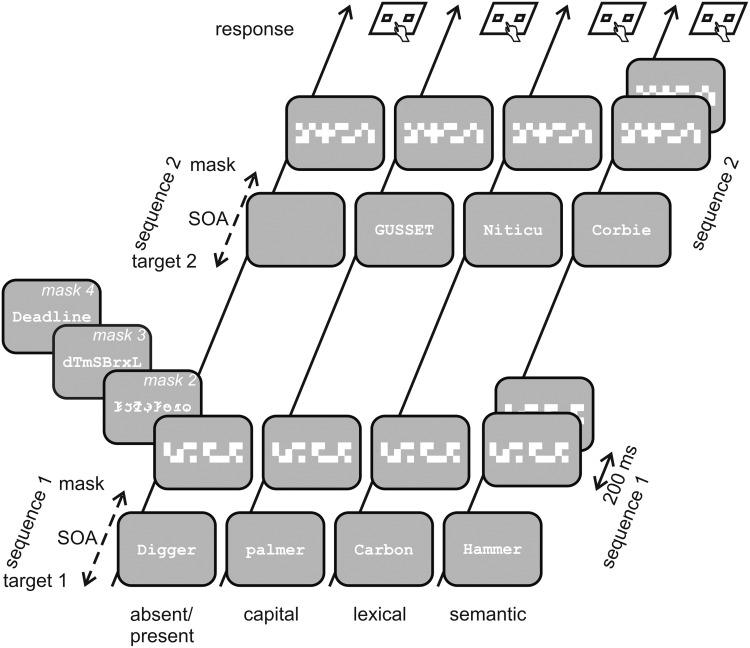
**Schematic drawing of the tasks.** Each of the four tasks consists of two target-mask sequences with target pairs (targets 1 and 2) representing the respective task features: word and blank screen, word in upper and lower cases, word and pseudoword, natural object and artifact. Duration of target presentation was one frame on the CRT screen. Stimulus onset asynchrony (SOA) between target and mask was identical in both target-mask sequences. It was adaptively varied in order to measure discrimination thresholds for the given task between 6.7 ms (no blank frame between target and mask) and 340 ms (50 blank frames between target and mask). Mask duration was 200 ms as depicted in the semantic condition, interval between the two target-mask sequences was 900 ms. Subjects had to indicate by button press in which of the two intervals the feature of the given task was seen (response). The four masks depicted as inset in interval 1 for the absent/present task (pattern, false font, random strings, and abstract words) were not varied within subjects but were administered to four different groups of subjects. The mask in interval 2 was always different to the mask in interval 1. The hash displayed at the begin of each interval and the question mark displayed following mask 2 are not depicted. Notice that text sizes and mask sizes are enlarged in relation to the screen for sake of clarity. The fifth task (visibility) was identical with the absent/present task, using only the shortest SOA of 6.7 ms.

Stimuli were generated using Psychopy (v1.78.01, cf. Peirce, 2007) and presented on a CRT screen (21′, iiyama, Hoofddorp, The Netherlands) at a frame rate of 150 Hz. Target words were flashed for one frame using the font Courier new with a height of 0.38° in white (25 cd/m^2^) on a gray background (5 cd/m^2^). According to the German spelling norms, first letters were always written in capital, with the exception in the capital task (see below). The mask with a duration of 30 frames (200 ms) followed the target with an SOA varying from 1 to 50 frames, i.e., 6.7–340 ms (**Figure 1**). Four different types of masks were applied: (a) a pattern mask consisting of 4 × 28 squares, either white (50 cd/m^2^) or gray (5 cd/m^2^, space averaged luminance 27.5 cd/m^2^) distributed at random, with a size of 3.5° by 0.5° covering the target words, (b) a random string of eight symbols from a false font created using elements of Courier new, (c) a random string of eight consonants, lower and upper cases mixed at random, (d) an abstract word, semantically unrelated to any of the target words, with a length of eight characters chosen at random from a list of 20 words, written with a capital at the beginning followed by lower case letters (**Figure 1**). Symbol string masks (b, c, and d) had the same height as target words (0.38°) and were two letters longer. Luminance of the three symbol string masks was 50 cd/m^2^. The string masks covered about 30% of the total space, resulting in a space averaged luminance of 18.5 cd/m^2^.

Subjects sat in front of a CRT screen with a distance of 1.5 m in a room with dimmed ambient light. During the experiment they responded with left and right index fingers, respectively, on a keyboard.

Since timing of the target – mask sequence was critical in the experiment, precision of sequences was tested externally using a photodiode at the CRT screen during development of the task. Test runs with variable SOAs demonstrated precise timing without frame drops and with stable durations of target and mask.

### Design of the Experiment

Participants performed five different two-interval forced choice tasks with 50 trials each, in which discrimination thresholds for a certain task feature were measured by adaptively varying the target-mask SOA. A trial consisted of the presentation of two target-mask sequences, finished by a temporal two-alternative forced choice decision. The sequence started with a hash (#) for 500 ms followed by a blank screen for 500 ms. Then the target word was flashed for one frame followed by the mask for 200 ms. Target and mask were separated by a blank period with a variable SOA of 6.7–340 ms. After a pause (blank screen for 900 ms following the presentation of the mask), the second sequence with identical timing parameters started with a hash. Thus, the second sequence started 2100 ms + SOA after the first sequence. Immediately after the end of the second sequence, a question mark indicated the subject to respond. (**Figure 1**, for sake of clarity the hash as well as the question mark are not depicted). As responding in this task is delayed and unconscious priming has been shown to decay rapidly after about 100 ms (Greenwald et al., 1996; Kiefer and Spitzer, 2000; Kiefer and Brendel, 2006), above-threshold performance in this temporal 2-AFC task most likely exclusively reflects awareness of the critical feature, because biases from unconscious processing are minimized (for a more detailed discussion of this issue, see the introductory section).

The order of the first four tasks was counterbalanced over subjects, followed by the 5th task, the visibility task, always at the end of the experiment. The five tasks were: (i) Absent/present task. In only one of the two target-mask sequences a word was flashed, while in the other sequence only the mask was shown. (ii) Capital task. In one sequence the target word was written in capitals, in the other sequence the target word was written in lower cases, including the first letter. (iii) Lexical decision task. A word or a pseudoword was presented as target in the sequences. (iv) Semantic task. One target word referred to a natural object, whereas the other target word named a man-made artifact. (v) Visibility task. Similar to the absent/present task, in only one sequence a target word was presented. In difference to the former task the target-mask SOA was fixed to 6.7 ms, and no discrimination threshold but a detection ratio was measured. This task served to assess, whether stimuli were entirely unconscious at minimal stimulus mask SOA.

Report categories (e.g., “In which interval was the word written in capital letters?” vs. “In which interval was the word written in lower case letters?”) were balanced over subjects. The target-mask interval, in which the critical stimulus was present (first or second interval), was varied randomly with the restriction that the critical stimulus appeared equally often in the first and second interval, respectively. Prior to each task, an instruction text on the screen described the task (e.g., “In one interval a word written in lower case letters will be presented, in the other interval the word will be written in capital letters.”) and the report category. Each task started with five training trials with a fixed SOA of 173 ms. Then 50 trials with adapting SOAs were presented. Two simple 2-down 1-up staircases were randomly intermixed, 25 trials each, to achieve a good sampling across a broad SOA range. The first staircase started at an SOA of 173 ms, step size was 33 ms. The second staircase started at an SOA of 73 ms, step size was 20 ms.

The effects of the four different masks were investigated in a between-subject design in order to keep the length of the entire experimental session (about 1 h) manageable for the participants. Furthermore, the number of available word stimuli for the semantic condition was too small to allow for presentation within a within-subject design without stimulus repetition. Each subject was randomly assigned to one of the four groups, and in each of the five tasks the same mask was used.

### Data Analysis

**FIGURE 2:**
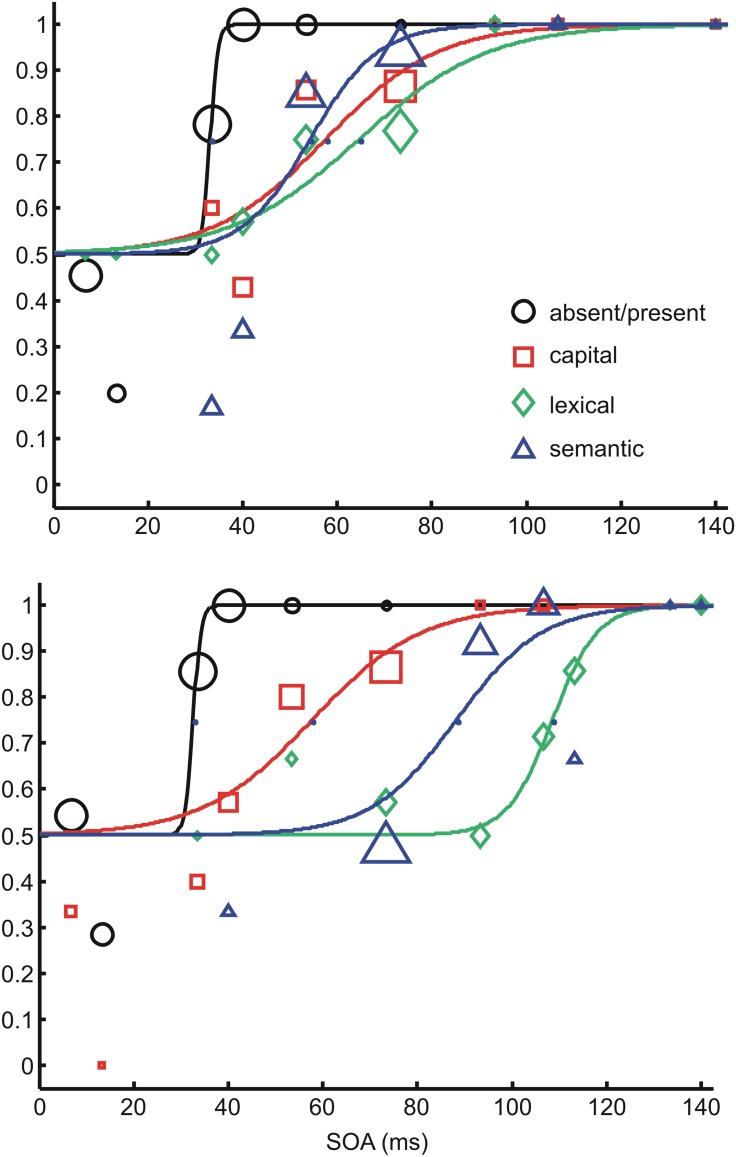
**Examples of psychometric functions fitted on data of two subjects in different masking conditions.** Top: participant in the pattern mask condition, bottom: participant in the word mask condition. The four different tasks are depicted in different symbols and different colors. The size of the symbols codes the number of presentations per SOA which differs due to the staircase procedures. In the examples the range of number of presentations is *n* = 1 (lower graph, red square at 17 ms) to *n* = 17 (lower graph, blue triangle at 73 ms). The largest SOA of 173 ms is not included into the graph for sake of clarity (performance 1.0 in each task). Slope values in the order of the data labels: upper 1.33, 0.0499, 0.0729, 0.139; lower 1.33, 0.0862, 0.227, 0.110.

In each individual and task, responses from the two staircases were collapsed, and a psychometric function (**Figure 2**) was fitted to the accuracy distribution as a function of target-mask SOA using psignifit (v2.5.6, cf. Wichmann and Hill, 2001). The logistic function

$$F(x)=\gamma +\frac{1-\gamma -\lambda }{1+e^{-\beta \left(x-\alpha \right)}}$$

with α as threshold, β as slope parameter, γ as probability rate, and λ as lapsus rate, was applied, with the free parameters α and β while fixing γ = 0.5 and λ = 0. Please notice that an increase in β yields a steeper psychometric function. Detection thresholds (absent/present task) or discrimination thresholds (the other three tasks) were defined as SOA with 75% correct responses (point of inflection of the logistic function, α).

Thresholds and slopes were analyzed using a mixed-design analysis of variance (ANOVA, Statistica V12, StatSoft, Hamburg, Germany). Greenhouse-Geyser corrections were applied in case of violation of sphericity (Mauchley’s test). We report Greenhouse-Geyser’s ε together with the uncorrected degrees of freedom. *Post hoc* analyses were performed using Newman–Keuls tests.

Seventeen subjects (20%) had to be excluded. In nine subjects, accuracy in the visibility task, in which masks were presented at the shortest possible target-mask SOA, was above chance performance [correct identification of 32/50 trials (64%) or more, above chance performance according to binomial distribution, *p* = 0.03]. We excluded these subjects from analyses of the main experiment, because it cannot be guaranteed that states of complete unawareness were reached, even at the shortest SOA. Performance above chance level at the shortest SOA does not allow a reliable threshold estimation based on fitting of a sigmoid function, because the lower left part of the function, performance at random, is not reached. In the remaining eight rejected subjects, thresholds could not be determined because the psychometric function of one or several tasks could not be adequately fitted due to a non-monotonous variation of performance as a function of target-mask SOA. The number of subjects included was: 17 (pattern mask), 17 (false font mask), 16 (random string mask), 17 (word mask).

## Results

### Analysis of Threshold

**FIGURE 3:**
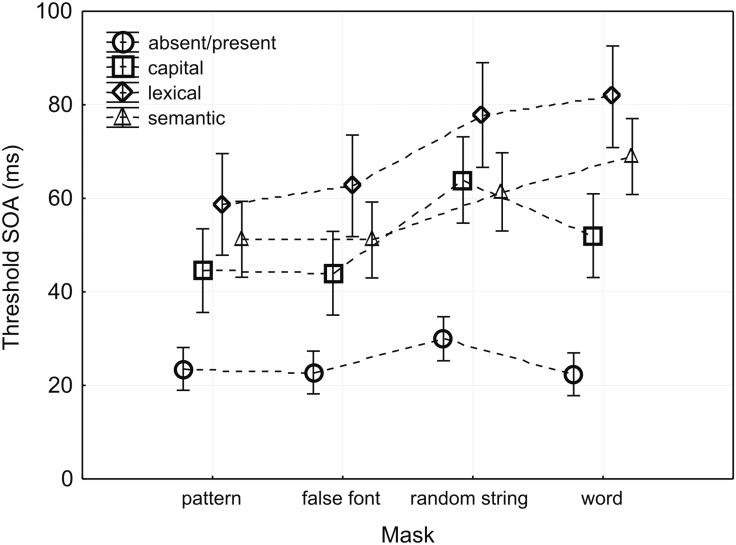
**Thresholds for each task (SOA in ms) in each of the four mask groups.** Depicted are mean values and the ±95% confidence interval. Please notice that dotted lines connecting the given task over the four different masks connect results from the four different groups.

Threshold data from 67 subjects were subjected to an omnibus mixed-design ANOVA with the group factor MASK (four groups: pattern, false font, random string, and word) and the within factor TASK (four levels: absent/present, capital, lexical, and semantic). Both factors revealed significant differences: group factor MASK [*F*(_3,63)_ = 5.0, *p* = 0.0036, $\eta_{p}^{2}$ = 0.19], within factor TASK [*F*_(3,189)_ = 145.4, ε = 0.90, *p* < 0.001, $\eta_{p}^{2}$ = 0.70]. Most importantly, the interaction MASK × TASK was also significant [*F*_(9,189)_ = 2.97, ε = 0.90, *p* = 0.0026, $\eta_{p}^{2}$ = 0.12] (**Figure 3** and Supplementary Table S2).

Regarding factor TASK, *post hoc* tests showed that lowest thresholds were obtained with the absent/present task, followed by capital and semantic tasks, whereas the lexical decision task resulted in highest thresholds (all comparisons *p* < 0.005). These *post hoc* tests thus revealed a gradient of awareness thresholds as a function of the task-relevant stimulus feature. *Post hoc* tests for the factor MASK indicated that thresholds were similar for mask 1 (pattern) and mask 2 (false font, *p* > 0.25). Thresholds for both masks were significantly lower compared to mask 3 (random string, pattern *p* = 0.019, false font *p* = 0.015) and mask 4 (word, pattern *p* = 0.033, false font *p* = 0.018). Thresholds for masks 3 and 4 did not differ from each other (*p* > 0.25).

In order to assess the complex interaction between the influences of MASK and TASK on awareness thresholds in more detail, subsidiary ANOVAs were calculated (**Table 1**). Firstly, between group analyses are reported comparing the impact of the four different masks on one of the four different tasks (**Table 1A** and **Figure 3**). In the absent/present task different masks did not significantly affect thresholds [between group comparison, *F*_(3,63)_ = 2.33, *p* = 0.083, $\eta_{p}^{2}$ = 0.10]. This shows that all masks were equally effective in interfering with the ability to consciously report the presence of a stimulus. For the remaining three tasks, capital, lexical, and semantic, the different masks significantly affected thresholds. In the capital task, mask 3 (random string) yielded significantly higher thresholds compared to mask 1 (pattern, *p* = 0.0097) and to mask 2 (false font, *p* = 0.014). Threshold with mask 3 (random string) was higher than that obtained with mask 4 [word; although this difference did not reach conventional significance levels (*p* = 0.066)]. Thresholds with mask 4 (word) did not significantly differ from those of mask 1 (pattern, *p* = 0.25) and mask 2 (false font, *p* > 0.25).

**Table 1:** Subsidiary analysis of variance (ANOVAs).

(A) Within a given task: effect of the between-subject factor mask.					

**Task**	**df**	***F***	***p***	**$\eta_{p}^{2}$**	
Absent/present	3,63	2.33	0.083	0.10	
Capital	3,63	4.14	0.0096	0.16	
Lexical	3,63	4.25	0.0085	0.17	
Semantic	3,63	4.52	0.0062	0.18	

**(B) Within a given masking group: effect of the within-subject factor task.**					

**Mask**	**df**	***F***	***p***	**ε (G–G)**	**$\eta_{p}^{2}$**

Pattern	3,48	26.8	<0.001	0.629	0.63
False font	3,48	37.7	<0.001		0.70
Random string	3,45	21.8	<0.001		0.59
Word	3,48	92.2	<0.001		0.85


A different pattern was observed for the two remaining tasks, lexical and semantic. In these higher-level tasks, mask 3 (random string) and in particular mask 4 (word) yielded the highest thresholds. In the lexical task, thresholds obtained with mask 3 (random string, *p* = 0.043) as well as with mask 4 (word, *p* = 0.021) were higher than thresholds with respect to mask 1 (pattern). Furthermore, the thresholds with mask 4 (word) were significantly higher than those of mask 2 (false font, *p* = 0.044). For mask 3 (random string), the difference to mask 2 (false fonts) just failed to reach conventional significance levels (*p* = 0.055). Finally, in the semantic task only thresholds obtained with mask 4 (word) were significantly higher compared to the thresholds obtained with mask 1 (pattern, *p* = 0.009) and mask 2 (false font, *p* = 0.016). Thresholds with mask 3 (random string) did not significantly differ from those of mask 1 (pattern, *p* = 0.085) and mask 2 (false font, *p* > 0.25) and those of the mask 4 (word, *p* = 0.20).

To summarize, the different tasks interfered differently with the different masks. In the absent/present task, thresholds did not differ across masks. Whereas in the capital task highest thresholds were obtained applying mask 3 (random string) followed by mask 4 (word), in the lexical and semantic task the highest thresholds were seen with mask 4 (word) and mask 3 (random string), although only thresholds with mask 4 consistently differed from both those of mask 1 (pattern) and mask 2 (false font). Thresholds with mask 1 (pattern) and mask 2 (false font) were always below mask 3 (random string) and mask 4 (word).

Secondly and most importantly to the purpose of the present study, we determined the threshold pattern of the different tasks within each masking condition. To this end, for each masking condition, a repeated-measure ANOVA with the within factor TASK was performed. For all masks, the factor TASK was significant (**Table 1B** and **Figure 3**). However, congruent with the interaction task × mask obtained in the omnibus ANOVA, the tasks elicited a differential pattern of thresholds depending on the masking condition. While the absent/present task yielded significantly lower thresholds compared to the other three tasks in all masks (all *p* < 0.001), the pattern of thresholds in the other tasks differed across masks. Thresholds in the capital and semantic tasks were comparable for mask 1 (pattern. *p* = 0.11), mask 2 (false font, *p* = 0.072) and mask 3 (random string, *p* > 0.25), but significantly differed from each other only with mask 4 (word, *p* < 0.001). Thresholds of the semantic task were comparable to those of the lexical task for mask 1 (pattern, *p* = 0.078). For the other masks, thresholds were consistently and significantly higher for the lexical than the semantic task (mask 2: *p* = 0.004, mask 3: *p* = 0.027, mask 4: *p* = 0.002). With all four masks, the capital task differed from the lexical task (mask 1: *p* = 0.004, mask 2: *p* < 0.001, mask 3: *p* = 0.028, mask 4: *p* < 0.001). To summarize, while the absent/present task consistently exhibited the lowest threshold across all masking conditions, the threshold pattern of the other tasks varied as a function of the mask. For mask 1 the thresholds for the capital, lexical and semantic tasks were least differentiated with only a significant difference between the capital and lexical tasks. For mask 4, in contrast, the threshold pattern was most differentiated with significant differences between all three tasks. The threshold pattern for masks 2 and 3 showed an intermediate form of differentiation with diverging thresholds for the lexical task on the one hand and those of the capital and semantic tasks on the other hand.

### Analysis of Slopes of the Psychometric Functions

**FIGURE 4:**
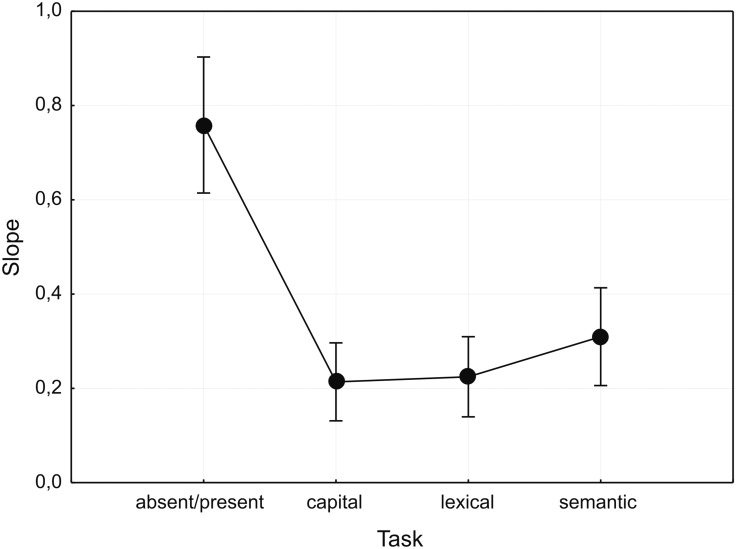
**Slopes of the psychometric functions, grouped by factor TASK.** Depicted are mean values ± 95% confidence interval.

Slopes of the fitted psychometric functions were analyzed (similar to threshold measures) by an omnibus mixed-design ANOVA with the group factor MASK (four groups: pattern, false font, random string, and word) and the within factor TASK (four levels: absent/present, capital, lexical, and semantic). Only the factor TASK yielded significant effects [*F*_(3,189)_ = 23.3, ε = 0.85, *p* < 0.001, $\eta_{p}^{2}$ = 0.27, **Figure 4** and Supplementary Table S3], whereas the group factor MASK [*F*(_3,63)_ = 0.073, *p* > 0.5, $\eta_{p}^{2}$ = 0.004] as well as the interaction MASK × TASK [*F*_(9,189)_ = 0.92, *p* > 0.5, *$\eta_{p}^{2}$* = 0.04] were not statistically significant. *Post hoc* tests showed that slopes for the absent/present task were steeper compared to the other three tasks (*p* < 0.001). The other task conditions did not differ from each other.

### Analysis of Visibility Task

Mean performance accuracy in the visibility task (absent/present at constant SOA of 6.7 ms) for all subjects included into the analysis (*n* = 67, see Materials and Methods) was 51.5% ± 0.62%, range from 38 to 62%. An ANOVA comparing the performance accuracy over the four mask groups revealed no systematic difference [*F*_(3,63)_ = 0.5, *p* > 0.25, $\eta_{p}^{2}$ = 0.02]. This suggests that masks were similarly effective at the smallest SOA.

## Discussion

The present study assessed the temporal emergence of visual awareness by determining the awareness thresholds of stimulus features of varying complexity using an objective psychophysical approach. In order to obtain a measure of the temporal dynamics of awareness, stimulus visibility was varied by manipulating target-mask SOA according to a staircase algorithm. Feature-specific discrimination thresholds were determined by fitting psychophysical functions to the accuracy distribution across trials as a function of target-mask SOA. Our approach has the advantage over detection measures based on signal detection theory that the fitted psychophysical function reflects the entire performance distribution and is not an average measure. Furthermore, our temporal 2-AFC choice task minimizes responses based on unconscious processing. Hence, in line with general psychophysical approaches to threshold measurements (Kingdom and Prins, 2016), our paradigm is suited to yield valid information about the temporal emergence of stimulus features contributing to a percept. We specifically tested whether the dispersion of feature discrimination thresholds depends on the context by varying the similarity between mask and stimulus. Such an interaction between feature type and mask would indicate a context-dependent dynamic emergence of visual awareness.

One might argue that psychophysical measures based on performance in masking experiments, e.g., thresholds as a function of the SOA as in our study, are not informative with regard to the emergence of visual awareness *per se*, but simply reflect resilience of different visual processes to mask interference or the ability of visual processes to handle two stimuli (target and mask) in parallel. We do not think that these alternative interpretations contradict our interpretation, because they interpret the data at a different theoretical level. Less interference by the mask should be related to a higher discrimination accuracy at shorter SOAs (i.e., a lower threshold), which in turn should index that phenomenal awareness of the stimulus feature is reached after shorter processing, i.e., temporally earlier. As we already have laid out in the section “Introduction,” in order to be able to make inferences from behavior to the content of phenomenal consciousness, we must make the plausible (but difficult to validate) assumption that phenomenal consciousness (subjective experience) and access consciousness (here: above-threshold discrimination performance) are at least correlated in time.

Furthermore, one could deny the assumption of a temporal correlation of threshold SOAs and the time course of the emergence of subjective experience. One could instead alternatively postulate that the time course of emergence into awareness is completely uncoupled from earlier processing stages we characterized by measuring threshold SOAs. This alternative assumption, however, seems implausible, since processing within neural networks typically occurs in a cascading fashion: Information from earlier processing stages is immediately transmitted to further stages so that temporal characteristics from earlier processing stages are still present or even more exaggerated at later stages (Humphreys et al., 1988; Kammer et al., 1999).

### Context-Dependency of Feature Thresholds: Emergence of Awareness across Feature Types

In line with our predictions, the type of the mask significantly influenced the relative sequence of feature thresholds SOAs. Although stimulus presence decisions (awareness of stimulus energy) had consistently the lowest threshold SOAs across masks, the differentiation of thresholds for the other features was strongly influenced by the specific masking context. For pattern masks, which do not show any similarity with any target feature, thresholds for the capital, lexical and semantic tasks were comparable with only a significant difference between the capital and lexical tasks. For the word mask, which has a high similarity with the target words at all levels of representation (energy, letter, lexical and semantic level), the temporal differentiation of feature thresholds was largest, with significant differences between all four tasks. Finally, the sequence of thresholds for the false font and random letters masks, respectively, which bear similarity with the target words only at the energy and letter level, showed an intermediate form of differentiation with diverging thresholds only for the lexical task on the one hand and those of the capital and semantic tasks on the other hand.

When considering the sequence of feature threshold SOAs, the least differentiated dispersion of thresholds under the pattern mask, although not compatible with the notion of a sharp dichotomous transition between unaware and aware states, suggests a less graded and more discontinuous emergence of awareness (Sergent and Dehaene, 2004; Sekar et al., 2013; Asplund et al., 2014): The absence-presence task had the lowest thresholds suggesting that stimulus presence (‘energy’) is rapidly consciously accessible. However, the more complex letter, lexical or semantic features exhibited significantly higher thresholds and thus contributed later, but almost simultaneously to the percept. In contrast, the high temporal differentiation of thresholds under the word masks suggest that awareness of letter, semantic and lexical word features emerges sequentially, consistent with both a gradual emergence of consciousness (Dennett and Kinsbourne, 1992; Overgaard et al., 2006; Seth et al., 2008) and the partial awareness hypothesis (Kouider et al., 2011; Windey et al., 2013; Windey and Cleeremans, 2015). The observed interaction between feature type and mask type shows that the temporal emergence of visual awareness is highly flexible (see also, Overgaard and Mogensen, 2016) and can be more discontinuous or gradual depending on the context (here mask type). Hence, these results support our proposal that the transition between unconscious and conscious visual perception can be characterized as reaching an attractor state within distributed brain areas (Herzog et al., 2016). Depending on the context, the attractor state might involve different visual and non-visual areas coding specific types of stimulus features at either comparable (discontinuous transition) or dispersed (graded transition) time points of visual processing.

In contrast to our and others’ (Kouider et al., 2011) predictions, lexical word features (lexical decisions) exhibited consistently across mask types (except for the pattern mask) higher thresholds than semantic word features (semantic decisions), although semantics is typically considered to be a higher representational level compared to lexical representations (McClelland and Rogers, 2003). Two mutually not excluding explanations of this unexpected finding are possible: (i) As pseudowords were created from real words by exchanging a few letters, a greater perceptual clarity is required to be able to successfully differentiate between words and pseudowords, in particular with similar masks. For semantic living/non-living decisions and the other tasks, the decision categories are better differentiated so that less perceptual clarity is needed. (ii) Awareness of word meaning might emerge earlier than awareness of lexicality (see also Anzulewicz et al., 2015) because semantic processing of the living/non-living dimension depends on an extended network of brain areas in fronto-parietal and occipital areas compared with lexical processing (Kiefer and Pulvermüller, 2012), which involves relative restricted areas in temporal cortex (Cohen and Dehaene, 2004). It is possible that activity within the larger semantic brain network accumulates faster within reverberating processing loops to reach the ignition level necessary for awareness (Noy et al., 2015).

### Context-Dependency of Slopes of the Psychophysical Functions: Emergence of Awareness within Features

In addition to feature threshold SOAs, we also analyzed the slopes of the fitted psychophysical function within each task: A shallow slope is taken to index a gradual transition from unawareness to awareness for a feature probed by a given task, whereas a steep slope indicates a discontinuous or even a sharp dichotomous transition (Koch and Preuschoff, 2007; Del Cul et al., 2009; Windey et al., 2013). Within the context of the partial awareness framework, one variant assumed an all-or-none emergence of awareness for single features, which should result in generally steep slopes (Kouider et al., 2011). According a second variant of the partial awareness framework, awareness of higher-level semantic features is assumed to be dichotomous resulting in relatively steeper slopes in contrast to the gradual emergence of awareness for lower-level perceptual color features leading to shallower slopes (Windey et al., 2013; Windey and Cleeremans, 2015). We further assumed that slopes for higher-level word and semantic features would be shallower for the random letter and word masks compared with pattern and false font masks, because random letter and word masks should specifically delay the processing of lexical and semantic word features.

In contrast to our or others’ expectations, we found the steepest slopes for the absent/present task independent of masking suggesting a rather discontinuous emergence of awareness. Slopes for the higher-level letter, lexical and semantic discrimination tasks were shallower than for the absent-present task and did not differ from each other. This suggests that for features more complex than the detection of stimulus presence (‘energy’) awareness seems to evolve more gradually.

When interpreting the slope parameter of a psychophysical function the following issues must be considered: Slopes cannot be classified as being “steep” or “shallow” without any reference, e.g., another psychophysical function with a different slope. However, in our view this holds true for any investigation in perceptual performance. We believe that any psychophysiological process evolves in time due to the functional properties of neurons within their networks. From that perspective, any perceptual process can be described with a transition function, which normally is sigmoidal. This formally contradicts the concept of a true step function, which might be the underlying concept of an “all-or-none” mechanism in perceptual awareness. However, a sigmoid function, where the transition between performance at chance level and performance without error happens within a few milliseconds, as for the absent/present task in the present study, looks like a step function. Such a very brief transition phase, although formally different from a true step function, could indicate a discontinuous emergence of awareness.

We are also aware that the slope value is related to the resolution of the abszissa and therefore, the first is influenced by the latter. Simulations with idealized data showed that using the present step size of 6.7 ms for the stimulus-mask SOA the steepest slope obtainable was 1.33. Decreasing the step size of the SOA to 2 ms (which is difficult to achieve with standard CRT monitors) would increase the steepest obtainable slope to 4.0. With respect to our results (steepest slopes in absent/present condition, mean slope value 0.759, Supplementary Table S3) we would therefore not expect a substantial change in the relation of the slope pattern in the present study. Of course, the magnitude of the slope can be characterized with more precision, when the step size of the SOA variation are smaller.

The pattern of slopes (steeper slope for energy than for higher-level features) was incompatible with our expectations and with earlier findings (Windey et al., 2013). We speculate that slopes of psychophysical functions and thus the transition from unawareness to awareness for a given feature are highly flexible and depend among other factors on the dimensional continuity of the features probed in the task (see also, Windey and Cleeremans, 2015): Dimensional continuity between blue and red hues, but also between living and non-living concepts is more continuous compared with stimulus absence vs. presence or with integer number magnitude, which are both intrinsically discontinuous. It is conceivable that awareness of a feature on a continuous dimension emerges rather gradually compared with features on discontinuous dimensions, which give rise to sharp transitions between unaware and aware states. As outlined in the introduction, the attractor state related to visual awareness might be reached from previous intermediate states of unconscious processing through relatively sharp (discontinuous feature dimension) or smooth (continuous feature dimension) transitions resulting in a more dichotomous or gradual emergence of awareness. Of course, this explanation is only tentative and has to be tested in future studies. Also contrary to our expectations, the slopes for lexical and semantic features were not modulated by the masks, i.e., they were not shallower for random letter and word masks, which are assumed to more strongly delay processing of these features compared with pattern and false font masks. It must remain open, whether the slopes for each feature type are relatively invariant and generally insensitive for influences of the different masks. Alternatively, it is possible that modulation of the slopes by the masks can be found under stimulation conditions, in which the influence of the mask is putatively stronger, for instance when the target stimuli are presented at lower contrasts.

## Conclusion

By using a psychophysical approach, the present study shows that the temporal transition between unaware and aware states is modulated by the context: Depending on the mask, word features (except energy) contribute almost simultaneously to the percept suggesting a more discontinuous transition from unawareness to awareness or had quite dispersed threshold SOAs indicating a highly temporally graded transition. A high similarity between the probed feature and the mask appears to be an important factor to induce a graded emergence of awareness across stimulus features. Analyses of slopes of the fitted psychophysical functions also indicated that the temporal emergence of awareness of single features is variable and might be influenced by the continuity of the feature dimensions. The present work thus suggests that the emergence of awareness is neither purely gradual nor dichotomous, but highly dynamic and context-dependent at both the stimulus and feature level. Future studies could combine psychophysical measurements of thresholds and slopes with subjective ratings of awareness in the search for convergent evidence regarding the temporal dynamics of visual awareness.

## Ethics Statement

The procedures of this study have been proved by the Ethics Committee of Ulm University. Participants received written information about the experimental procedure and signed a written informed consent.

## Author Contributions

MK and TK developed the study concept and were involved in data collection. TK performed the data analyses in cooperation with MK. MK and TK drafted the manuscript, provided critical revisions and approved the final version for submission.

## Conflict of Interest Statement

The authors declare that the research was conducted in the absence of any commercial or financial relationships that could be construed as a potential conflict of interest.
