# Supplementary material for: The Emergence of Visual Awareness: Temporal Dynamics in Relation to Task and Mask Type
Source: Front Psychol. 2017 Mar 3;8:315. doi: 10.3389/fpsyg.2017.00315 (PMC5334328; doi:10.3389/fpsyg.2017.00315)
Supplement: Supplementary file 1 [file Table_1.DOCX]

Supplementary Table S1

word lists

absent / present (single words)

| Obacht | Obdach | Borste | Gepard | Metier |
| --- | --- | --- | --- | --- |
| Morast | Hoftor | Imbiss | Pardon | Razzia |
| Gebein | Gigant | Schaum | Frosch | Grippe |
| Rubrik | Rummel | Orient | Tausch | Athlet |
| Kissen | Profit | Zirkus | Stange | Zusatz |
| Umlauf | Dusche | Ehrung | Ritter | Gitter |
| Bronze | Trikot | Budget | Statur | Rosine |
| Zirkel | Spende | Steppe | Brille | Statik |
| Kuchen | Studio | Spruch | Klappe | Walzer |
| Lektor | Heizer | Beirat | Skizze | Palais |

absent / present – test on visibility (single words)

| Hopfen | Karren | Lerche | Rechen | Parfum |
| --- | --- | --- | --- | --- |
| Kobalt | Fossil | Frevel | Garant | Gehege |
| Ritual | Wehmut | Embryo | Plasma | Radius |
| Siegel | Tribun | Zufuhr | Zulage | Marsch |
| Magnet | Pointe | Neptun | Zeiger | Utopie |
| Vakuum | Fessel | Fracht | Vorort | Gehirn |
| Gesuch | Belang | Gewand | Revier | Tempel |
| Effekt | Heirat | Status | Butter | Affekt |
| Umlauf | Mieter | Pathos | Zettel | Pastor |
| Freske | Abflug | Makler | Sparer | Achsel |

Capital (pairs of words)

| ARKADE | diesel | ARZNEI | eigelb | ANGLER | nomade |
| --- | --- | --- | --- | --- | --- |
| ASTHMA | nymphe | FLOSSE | schabe | FUNKER | schlot |
| MANEGE | glatze | MENTOR | brause | SPANGE | geiger |
| WINDEL | granat | FRIESE | spesen | GEMACH | steige |
| DEKRET | balkan | FURCHE | banner | KONSUL | abteil |
| LAWINE | akteur | RETTER | kneipe | SCHERE | lenker |
| KANONE | perser | KNOTEN | pilger | PATENT | rausch |
| ROMANE | rheuma | SCHILD | flanke | SCHIRM | fliese |
| MERKUR | traber | RIVALE | tunnel | SPERRE | stapel |
| TURNER | archiv | VULKAN | vetter | WIRTIN | gerede |
| IRONIE | diener | SEMMEL | dozent | MAURER | sattel |
| PAROLE | flagge | KNECHT | ladung | EREMIT | ostern |
| MANDAT | umkehr | REGION | diesel | KLINKE | klette |
| GEBELL | lineal | ABFALL | anreiz | ABSAGE | poetik |
| PLANET | rektor | TALENT | wandel | OPTION | kosmos |
| PRACHT | justiz | EXTREM | safari | MOSAIK | abbild |
| KURIER | trumpf | LIZENZ | junker |  |  |

Lexical decision (pairs of words)

| Zauber | Gpacht | Speise | Kantop | Pforte | Phyris |
| --- | --- | --- | --- | --- | --- |
| Koppel | Tsopen | Murmel | Pakzer | Pulver | Ekzoss |
| Muskel | Kommel | Kapsel | Possee | Weiche | Kiltus |
| Zement | Pkonem | Ketzer | Glofia | Geisel | Taimme |
| Knospe | Nikbus | Starre | Chopal | Krippe | Werler |
| Tonart | Pertie | Gehalt | Urnild | Akkord | Vorxof |
| Orakel | Unzakl | Denker | Moxile | Erdgas | Mafime |
| Nichte | Dremer | Ordner | Klisis | Schale | Tamkon |
| Diplom | Pakron | Kasten | Phrale | Braten | Mytgos |
| Segler | Fibaro | Balkan | Pletin | Banner | Miliku |
| Askese | Wajner | Nenner | Gultan | Nische | Kolist |
| Schnur | Gepade | Schutt | Pkofil | Messer | Wietne |
| Scheck | Fawtum | Taktik | Reshen | Ziffer | Davise |
| Wagnis | Temmor | Dichte | Vifion | Bandit | Fusiog |
| Schatz | Diamog | Dunkel | Pfloge | Lachen | Rezegt |
| Tugend | Atbung | Gipfel | Sanode | Marmor | Manaer |
| Meteor | Slreif | Grafik | Fedium |  |  |

Semantic decision (pairs of words)

| Ananas | Anorak | Reiher | Bohrer | Natter | Quader |
| --- | --- | --- | --- | --- | --- |
| Krabbe | Tresor | Wipfel | Gondel | Kaktus | Prisma |
| Ferkel | Pendel | Zander | Pfanne | Pappel | Podest |
| Falter | Pyjama | Melone | Spaten | Möhren | Beutel |
| Hammel | Pinsel | Hummer | Wimpel | Kiefer | Bikini |
| Traube | Bagger | Spinat | Ballon | Distel | Klinge |
| Schilf | Strick | Ameise | Galgen | Tomate | Kerker |
| Storch | Portal | Banane | Tapete | Eiland | Ziegel |
| Hengst | Becher | Insekt | Balken | Urwald | Bonbon |
| Hering | Statue | Hirsch | Tanker | Schwan | Reifen |
| Gerste | Karton | Jaguar | Kognak | Fliege | Whisky |
| Roggen | Messer | Frucht | Waggon | Weizen | Bunker |
| Auster | Teller | Fohlen | Pfeife | Grille | Hammer |
| Reptil | Brille | Schnee | Sessel | Wurzel | Gewehr |
| Spinne | Umhang | Donner | Splitt | Wimper | Perlon |
| Arktis | Rohbau | Mandel | Schild | Wermut | Plakat |
| Getier | Klinik | Kaviar | Bomber |  |  |
