# Supplementary material for: The Emergence of Visual Awareness: Temporal Dynamics in Relation to Task and Mask Type
Source: Front Psychol. 2017 Mar 3;8:315. doi: 10.3389/fpsyg.2017.00315 (PMC5334328; doi:10.3389/fpsyg.2017.00315)
Supplement: Supplementary file 2 [file Table_2.DOCX]

Supplementary Table S2

Mean SOA as a function of task and mask

| Mask | Task | mean SOA  (ms) | ±95% confidence  (ms) | n |
| --- | --- | --- | --- | --- |
| pattern | absent/present | 23.5 | 19.0 - 28.1 | 17 |
|  | capital | 44.5 | 35.6 - 53.5 |  |
|  | lexical | 58.7 | 47.8 - 69.6 |  |
|  | semantic | 51.3 | 43.1 - 59.4 |  |
| false font | absent/present | 22.8 | 18.2 - 27.4 | 17 |
|  | capital | 44.0 | 35.0 - 52.9 |  |
|  | lexical | 62.7 | 51.8 - 73.5 |  |
|  | semantic | 51.1 | 43.0 - 59.2 |  |
| random string | absent/present | 30.0 | 25.3 - 34.7 | 16 |
|  | capital | 63.9 | 54.7 - 73.2 |  |
|  | lexical | 77.8 | 66.6 - 89.0 |  |
|  | semantic | 61.4 | 53.0 - 69.7 |  |
| word | absent/present | 22.4 | 17.8 - 27.0 | 17 |
|  | capital | 52.0 | 43.1 - 61.0 |  |
|  | lexical | 81.7 | 70.9 - 92.6 |  |
|  | semantic | 69.0 | 60.8 - 77.1 |  |
