# Supplementary material for: The Emergence of Visual Awareness: Temporal Dynamics in Relation to Task and Mask Type
Source: Front Psychol. 2017 Mar 3;8:315. doi: 10.3389/fpsyg.2017.00315 (PMC5334328; doi:10.3389/fpsyg.2017.00315)
Supplement: Supplementary file 3 [file Table_3.DOCX]

Supplementary Table S3

Mean slopes as a function of task (n=67)

| Task | mean slope | ±95% confidence |
| --- | --- | --- |
| absent/present | 0.759 | 0.614 – 0.903 |
| capital | 0.214 | 0.131 – 0.297 |
| lexical | 0.225 | 0.140 – 0.310 |
| semantic | 0.310 | 0.206 – 0.413 |
